# Effect of food source availability in the salivary gland transcriptome of the unique burying beetle *Nicrophorus pustulatus* (Coleoptera: Silphidae)

**DOI:** 10.1371/journal.pone.0255660

**Published:** 2021-09-23

**Authors:** Christian O. Ayala-Ortiz, Jacob W. Farriester, Carrie J. Pratt, Anna K. Goldkamp, Jessica Matts, W. Wyatt Hoback, John E. Gustafson, Darren E. Hagen

**Affiliations:** 1 Department of Animal and Food Science, Oklahoma State University, Stillwater, Oklahoma, United States of America; 2 Department of Entomology and Plant Pathology, Oklahoma State University, Stillwater, Oklahoma, United States of America; 3 Department of Biochemistry and Molecular Biology, Oklahoma State University, Stillwater, Oklahoma, United States of America; Youngstown State University, UNITED STATES

## Abstract

*Nicrophorus* is a genus of beetles that bury and transform small vertebrate carcasses into a brood ball coated with their oral and anal secretions to prevent decay and that will serve as a food source for their young. *Nicrophorus pustulatus* is an unusual species with the ability to overtake brood of other burying beetles and whose secretions, unlike other *Nicrophorus* species, has been reported not to exhibit antimicrobial properties. This work aims to better understand how the presence or absence of a food source influences the expression of genes involved in the feeding process of *N*. *pustulatus*. To achieve that, total RNA was extracted from pooled samples of salivary gland tissue from *N*. *pustulatus* and sequenced using an Illumina platform. The resulting reads were used to assemble a *de novo* transcriptome using Trinity. Duplicates with more than 95% similarity were removed to obtain a “unigene” set. Annotation of the unigene set was done using the Trinotate pipeline. Transcript abundance was determined using Kallisto and differential gene expression analysis was performed using edgeR. A total of 651 genes were found to be differentially expressed, including 390 upregulated and 261 downregulated genes in fed insects compared to starved. Several genes upregulated in fed beetles are associated with the insect immune response and detoxification processes with only one transcript encoding for the antimicrobial peptide (AMP) defensin. These results confirm that *N*. *pustulatus* does not upregulate the production of genes encoding AMPs during feeding. This study provides a snapshot of the changes in gene expression in the salivary glands of *N*. *pustulatus* following feeding while providing a well described transcriptome for the further analysis of this unique burying beetle.

## Introduction

Carrion beetles (Coleoptera, Silphidae) have been widely studied by ecologists as they are among the first insects to arrive at vertebrate carrion and display a number of interesting behaviors to utilize this ephemeral resource [1]. Most members of the genus *Nicrophorus* (burying beetles), bury appropriately-sized vertebrate carcasses and transform them into a brood ball, which will serve as a food source for offspring [2]. For burying beetles, access to carrion is necessary for reproduction since copulation only occurs after the food source has been buried. Thus, these insects have evolved strategies and behaviors to take advantage of the unpredictable availability of this resource [2, 3].

Burying beetles are unusual in providing a high degree of bi-parental care to their offspring [2, 4]. They defend vertebrate carcasses, coat them with oral or anal secretions to retard decay [5, 6], and the parents regurgitate pre-digested carrion into the mouths of their young [7]. The oral and anal secretions of these beetles reduce carcass degradation [5, 8], and inhibit the growth of yeast and bacteria that are found within the burying beetles habitat and may act as competitors for the nutritious buried carcasses [9].

Research on *Nicrophorus orbicollis*, *Nicrophorus investigator* and *Nicrophorus marginatus* has confirmed the presence of antimicrobial peptides (AMP) in oral and anal exudates [9]. Chemical analyses of anal and oral secretions of *Nicrophorus vespilloides* found these exudates contain more than 30 secondary metabolites [10] and bacterial cell wall active lysozymes [11].

A study in *N*. *vespilloides*, using whole-adult RNA samples and anal exudates, reported the expression of genes that encode for 27 putative antimicrobial peptides (AMPs) and 13 lysozymes [12]. Another study in *Nicrophorus carolinus* [13] demonstrated that the antimicrobial properties exhibited from secretion were temperature-dependent and expressed during warmer temperatures.

The burying beetle *Nicrophorus pustulatus* is an unusual species in that exhibits the ability to overtake brood of other burying beetles and has been reported to produce secretions that do not exhibit antimicrobial properties [14]. In nature, unlike other *Nicrophorus* species, *N*. *pustulatus* has been reported to use snake eggs instead of vertebrate carcasses for breeding [15–17]. Similar to other carrion beetles, *N*. *pustulatus* feeds on vertebrate carrion and live insects when not breeding, using extra oral digestion and ingesting the digested fluids [18]. Observations of adult and larvae *N*. *pustulatus* in the nests and eggs of the black rat snake, *Elaphe obsoleta*, indicate a host shift from carrion to snake egg parasitoidism [15]. Laboratory studies confirmed that *N*. *pustulatus* efficiently exploit snake eggs and treat them differently from carrion, not moving or burying the eggs [16]. The communal nesting behavior and clutch size of black rat snakes also provides a large resource of *N*. *pustulatus*, perhaps explaining why it produces the largest broods compared to congeners in captivity [2]. Despite having never buried any of 1000+ research carcasses in the field [2], in the laboratory *N*. *pustulatus* can still use vertebrate carcasses for reproduction [17, 19].

Although there are some molecular resources for the *Nicrophorus* beetles, most of the available transcriptomic data has been generated for *N*. *vespilloides* [20, 21] and *N*. *orbicollis* [22, 23]. We now report on the salivary gland RNA-seq generated transcriptomes of male and female *N*. *pustulatus* following starvation and feeding. We hypothesized that the presence or absence of a food source will influence the expression of salivary gland genes involved with the feeding process in *N*. *pustulatus*. The transcriptomes produced also provide a resource that will support further investigations on the transcriptional responses of the *Nicrophorus*.

## Materials and methods

### Insect collection and rearing

Beetles were collected as previously described [24] in May 2018 on Camp Gruber Training Center in Muskogee County, OK. The beetles were collected as part of a study on the federally protected *Nicrophorus americanus* under authority of federal permit # TE045150-3 held by Hoback. The *N*. *pustulatus* used in the present study are not a protected species. Beetles were collected with permission of the Oklahoma Army National Guard. Insects were trapped using 5-gallon above-ground Silphidae bucket traps filled with approximately 7cm of peat moss and baited with a rotted rat carcass (RodentPro.com, IN) [25]. Captured beetles were identified based on morphology and kept alive in moist soil, half of them were deprived of food and half of them were provided high-fat (30%) ground beef as sustenance. After 5 days, beetles were anesthetized in an ice bath before the salivary gland tissue was removed for RNA extraction.

### RNA isolation and library preparation

RNA extraction was performed for a total of six biological replicates (3 pools of males and 3 pools of females) for each fed or starved conditions. Each biological replicate consisted of the combined salivary gland tissue of five individual beetles of the same sex. The combined salivary gland tissue was submerged in a tube containing RNAzol RT (Molecular Research Center, Inc. Cincinnati, Ohio), frozen with liquid nitrogen, and stored at -80°C. Total RNA was isolated from homogenized salivary gland tissue using RNAzol RT according to the manufacturers protocol (ThermoFisher Scientific). Briefly, tissues were homogenized in a microfuge tube with a plastic pestle in 500 μl RNAzol RT and allowed to stand at 25°C for 5 min. After chloroform addition (200 μl), samples were vigorously mixed for 2 min followed by centrifugation (13,000 X g for 10 min). The aqueous layer was then placed in a microfuge tube and an equal volume of isopropanol was added, and after mixing, the samples were placed at -20°C (16 h). The samples were then centrifuged (13,000 X g for 30 min) and the pellets were washed once with 75% ethanol and once with 70% ethanol, and then re-pelleted (13,000 X g for 5 min). The supernatant was removed, and the dried pellets were re-suspended in nuclease-free water. RNA samples were quantified with a NanoDrop (ThermoFisher, MA) and size distributions analyzed by BioAnalyzer 2100 (Agilent, CA) using a RNA Nano chip.

One microgram of RNA samples from each sample was used to construct Illumina TruSeq RNA Sample Preparation Kit v2 libraries following the manufacturer’s instructions (Illumina Inc., San Diego, California), except only 13 PCR amplification cycles were conducted. The Illumina libraries were quantified and quality was assessed using the BioAnalyzer 2100 DNA 100 chip. All libraries had similar size distributions between 200–500 bp with a ~260 bp peak. The libraries were sent to Novogene (Davis, California) for paired-end RNA sequencing.

### Transcriptome *de novo* assembly and annotation

Read quality was assessed using FastQC (version 0.11.3) [26] and adapters and low quality bases were trimmed using Trimmomatic (version 0.38) [27]. A *de novo* transcriptome assembly was generated by Trinity (version 2.8.1) [28] using merged reads from all libraries. A unigene set was obtained from the transcriptome by removing sequences with over 95% similarity using CD-HIT (version 4.8.1) [29, 30]. Assembly quality and completeness of both the transcriptome and the unigene set was assessed using BUSCO (version 4) with the Arthropoda ortholog dataset [31, 32].

### Differential expression analysis

Transcript abundance was determined using the pseudo-aligner Kallisto (version 0.46.2) [33] with the unigene set. Differential gene expression analysis between libraries of fed and starved *N*. *pustulatus* was performed in R (version 4.0.0) [34] using the edgeR package (version 3.30) [35, 36]. Read counts were normalized using the trimmed mean of M-values method [37]. Differentially expressed genes (DEGs) were defined using the ‘robust’ algorithm of edgeR using False Discovery Rate (FDR) to adjust for multiple testing [38] and determine significance.

### Functional annotation and pathway analysis

Transcriptomes were annotated using the Trinotate pipeline, a comprehensive annotation tool that uses well referenced methods and databases to generate functional annotation of *de novo* assembled transcriptomes [39]. For annotation, *in silico* peptide sequences were obtained using Transdecoder (version 5.5.0) [39]. Annotation of the transcriptomes included homology searches against the UniProtKB/Swiss-Prot database [40] and the NCBI ref-seq database [41] using BLAST+ (version 2.8.1) [42]. Protein domains in the peptide sequences were identified using HMMER version 3.1b2 [43] to query against the Pfam database [44]. Ribosomal RNA (rRNA) genes were identified in the transcriptomes using RNAmmer (version 1.2) [45].

Gene Ontology (GO) terms were obtained from the annotation pipelines against the Swiss-Prot database. KEGG Orthology (KO) terms were assigned using the KEGG Automatic Annotation Server (KAAS) [46]. Gene set enrichment analysis (GSEA) of the GO terms was done in R using the package topGO (version 2.40.0) [47] by comparing the assigned GO terms of the differentially expressed genes to the GO terms of the whole unigene set.

For the KO terms, gene set enrichment analysis was performed using the R package clusterProfiler [48] and comparing the assigned KO terms of the differentially expressed genes to a section of the universe of KO terms in the KO database. The section of KO terms used for the analysis corresponded to those that were assigned to pathways present in *Nicrophorus vespilloides* [49].

### Availability of supporting data

Raw RNA-Seq data is deposited in FASTQ format to the NCBI Sequence Read Archive database (SRA) and assembled transcripts to the NCBI Transcriptome Shotgun Assembly (TSA) Database under the BioProject accession number PRJNA740345. Code and project-specific scripts detailing parameters used for all analysis performed in this study can be found in the GitHub Repository: https://github.com/Coayala/npustulatus.

## Results

### Transcriptome assembly

The transcriptome of *N*. *pustulatus* was assembled using pooled reads from all libraries of the different experimental conditions. The resulting assembly produced a total of 78,390 transcripts. A unigene assembly set was created by reducing transcripts sharing more than 95% similarity to a single representative, resulting in a final assembly of 61,290 contigs. The statistics of the whole transcriptome assembly and the unigene are detailed in Table 1.

**Table 1:** Assembly statistics.

	Trinity Assembly	Unigene set
Total assembled bases	135,410,906	84,714,160
Number of contigs	78,390	61,290
Average contig length	1,727	1,382
N50	3,800	3,290
GC content (%)	40.10	39.92

**Fig 1:**
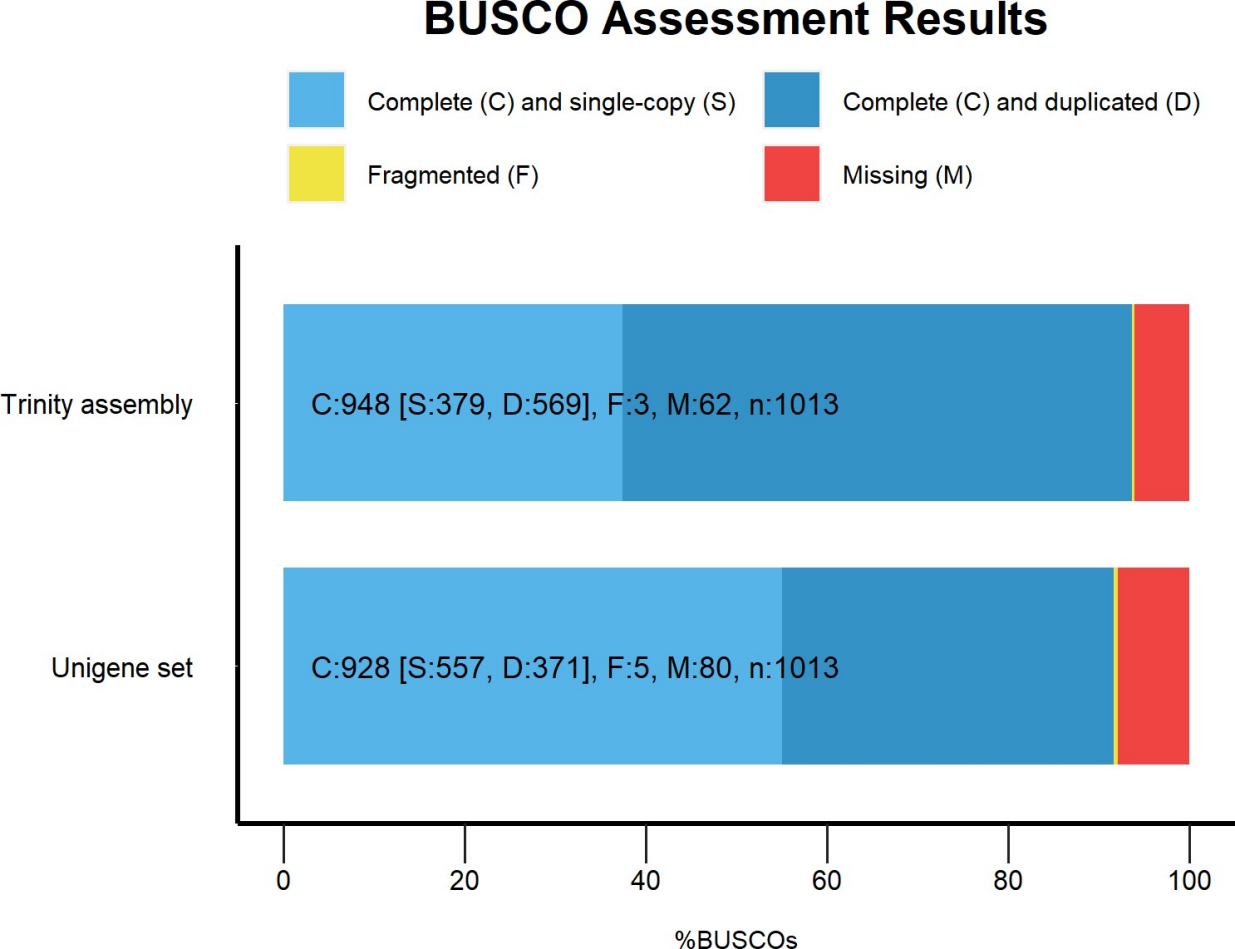
BUSCO completeness assessment. Both the transcriptome and the unigene set are more than 90% complete. Duplication levels are lower in the unigene set.

Completeness of the assembly and unigene set was evaluated using BUSCO [31, 32] with the Arthropoda dataset. The transcriptome and the unigene dataset were found to contain more than 91% of the single-copy orthologs in the dataset (Fig 1), and thus our transcript data is fairly complete.

### Transcriptome annotation

The unigene transcriptome was annotated using the Trinotate pipeline and representative peptide sequences using Transdecoder [39]. The nucleotide and peptide sequences of *N*. *pustulatus* were searched against three different databases, the Uniprot/Swiss-prot database [40], the NCBI RefSeq database [41] and the Pfam database [44]. In total, 65.70% of transcripts in the unigene set produced a significant hit against the NCBI RefSeq database and only 38.69% of them produced significant hits against the Uniprot/Swissprot database (Table 2).

**Table 2:** Number and percentage of significant hits against biological relevant databases.

	NCBI RefSeq	Uniprot/Swiss-prot	Pfam
Number of contigs with hits	40,270	23,713	26,689
Percentage of contigs with hits (%)	65.70	38.69	43.54

**Fig 2:**
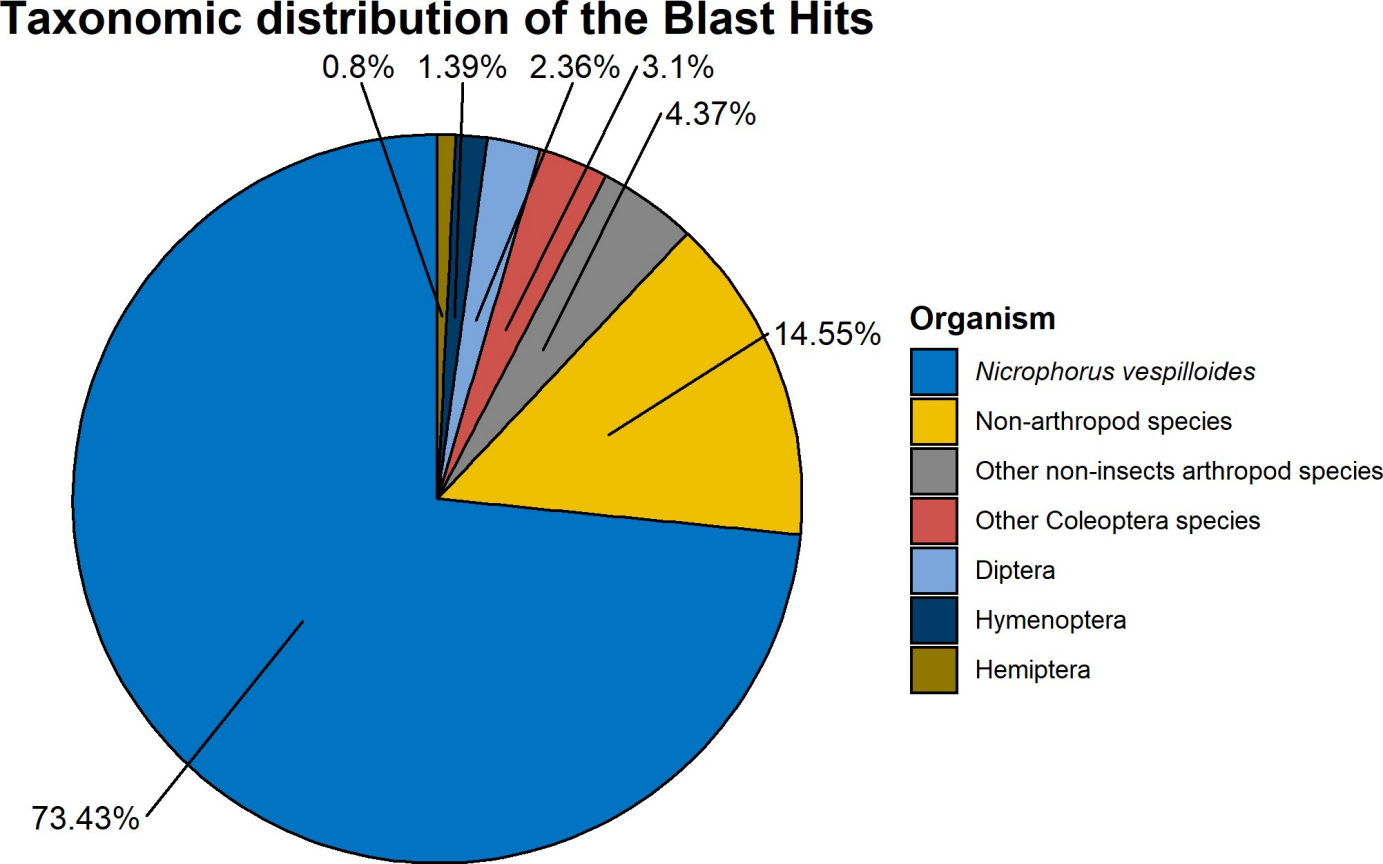
Taxonomic distribution of the Blast hits of the unigene set.

During the annotation, over 73% of the annotated unigenes produced a significant hit against reference sequences of the closely related species *N*. *vespilloides*, and to a lesser extent, other Insecta species as well (Fig 2).

### Differential gene expression

**Fig 3:**
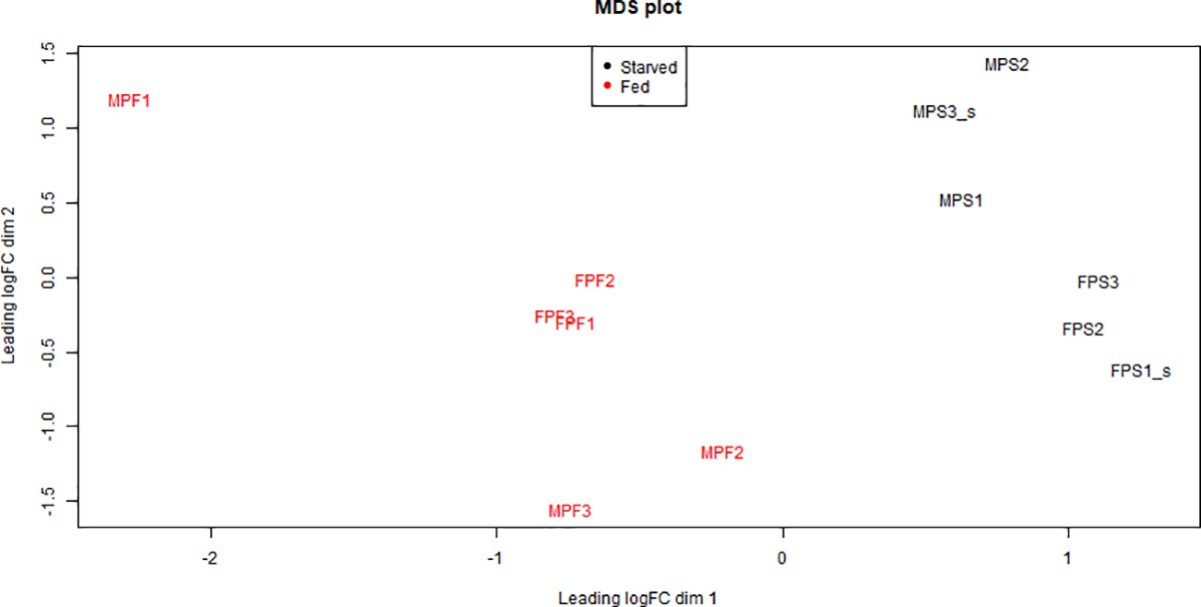
Multi-dimensional scaling (MDS) plot of the normalized read counts. Samples cluster together based on whether they came from a fed or starved beetle.

A differential gene expression (DGE) analysis of the transcriptomes obtained from the salivary glands of fed and starved *N*. *pustulatus* was performed in order to identify genes that altered their expression following feeding in this species. After pseudo-aligning the reads to the unigene set using Kallisto [33], and filtering lowly expressed transcripts, the remaining 13,555 transcripts were analyzed using edgeR [36]. A multi-dimensional scaling (MDS) plot shows the samples cluster together primarily by treatment (fed vs starved) and secondarily by sex (Fig 3). For this reason, it was decided to perform the following analysis by comparing only between treatments and not between sexes.

**Fig 4:**
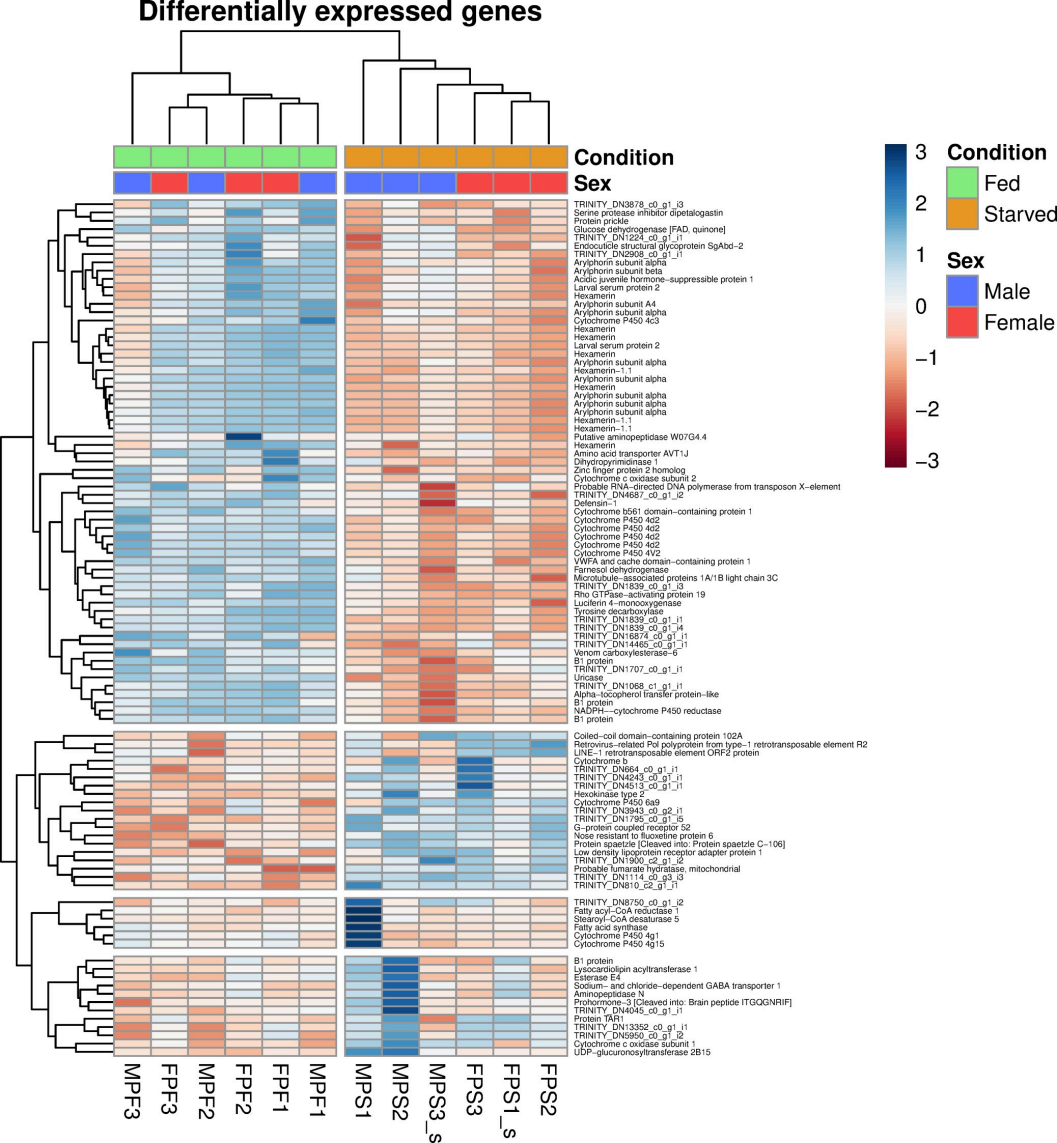
Heatmap of differentially expressed genes. The expression levels are quantified as log-transformed counts-per-million. The names of the genes were obtained from the annotation with the NCBI database.

After analysis, 651 significant (FDR < 0.05) DGEs were identified. Of those, 390 genes were upregulated in the fed insects while the remaining 261 were downregulated. A heatmap of differentially expressed genes with the greatest log fold change (logFC) (FDR <0.05 and |logFC| > 2) can be seen in Fig 4.

### Gene Ontology and KEGG pathway enrichment analysis

**Fig 5:**
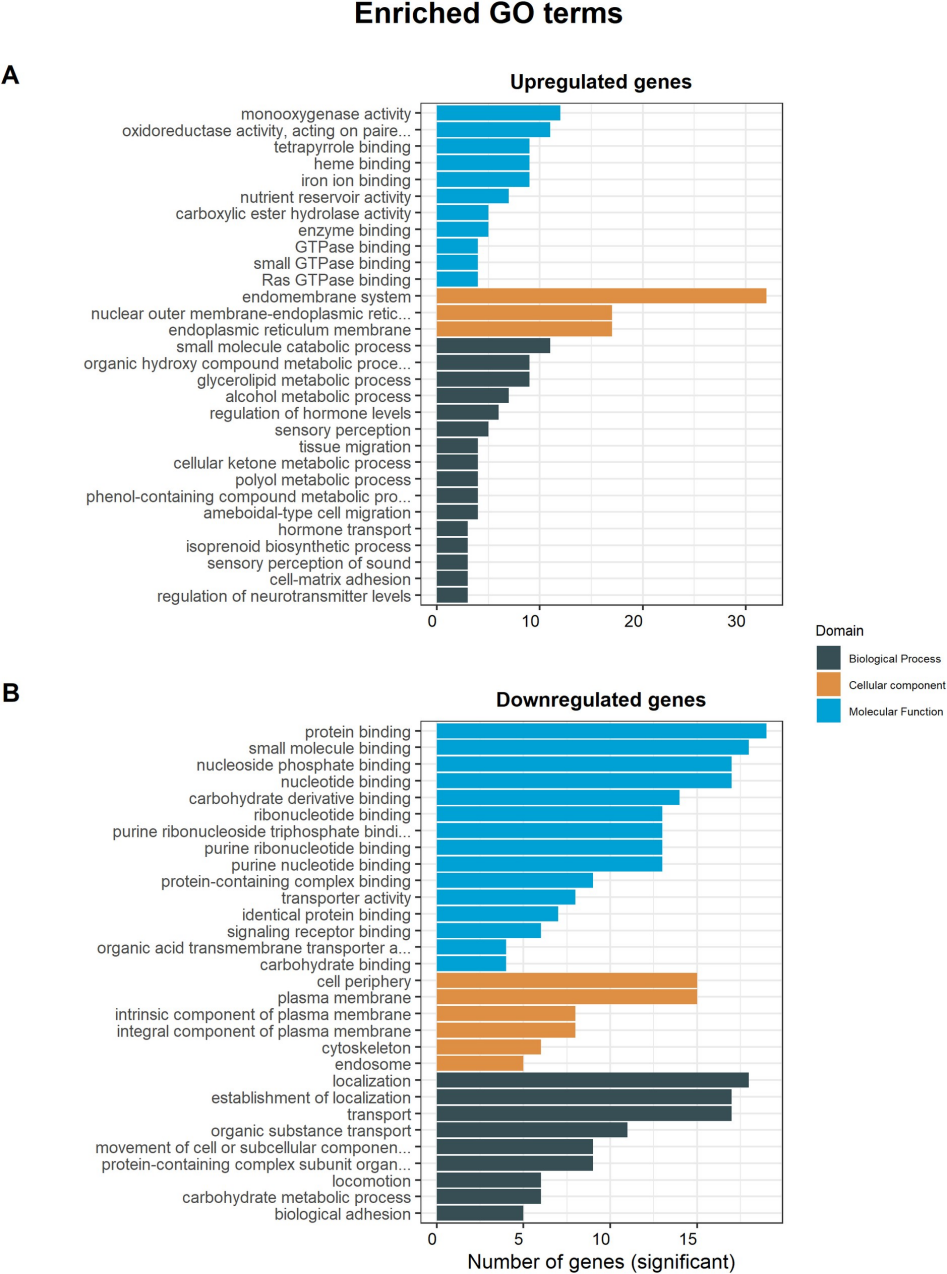
Enriched GO terms in the set of differentially expressed genes. (A) Enriched GO terms among the upregulated genes in the fed beetles. (B) Enriched GO terms among the downregulated genes in the fed beetles.

To better understand the molecular and biological responses of *N*. *pustulatus* to feeding and starvation, topGO [47] was used to perform a GSEA and identify enriched GO terms in the differentially expressed genes. Enriched GO terms were ranked based on the significance of their enrichment for each domain. Table 3 shows the top significantly enriched GO terms among all the DEGs, while Fig 5 shows the significantly enriched GO terms among the upregulated (Fig 5A) and downregulated genes in fed beetles (Fig 5B).

**Table 3:** Enriched GO terms in the set of 651 differentially expressed genes.

GO.ID	Term	# of significant genes	Rank
**Biological Process**			
GO:0071702	organic substance transport	4	6
GO:0006865	amino acid transport	3	1
GO:0006820	anion transport	3	2
GO:0015711	organic anion transport	3	3
GO:0015849	organic acid transport	3	4
GO:0046942	carboxylic acid transport	3	5
GO:0000495	box H/ACA snoRNA 3’-end processing	1	7
GO:0001659	temperature homeostasis	1	8
GO:0001678	cellular glucose homeostasis	1	9
GO:0001710	mesodermal cell fate commitment	1	10
GO:0003416	endochondral bone growth	1	11
GO:0019725	cellular homeostasis	1	12
GO:0033500	carbohydrate homeostasis	1	13
GO:0033979	box H/ACA snoRNA metabolic process	1	14
GO:0034964	box H/ACA snoRNA processing	1	15
GO:0035265	organ growth	1	16
GO:0042593	glucose homeostasis	1	17
GO:0048333	mesodermal cell differentiation	1	18
**Cellular component**			
GO:0005576	extracellular region	13	6
GO:0005615	extracellular space	7	1
GO:0005886	plasma membrane	7	19
GO:0005887	integral component of plasma membrane	2	13
GO:0031226	intrinsic component of plasma membrane	2	14
**Molecular Function**			
GO:0004497	monooxygenase activity	8	2
GO:0045735	nutrient reservoir activity	5	1
GO:0000010	trans-hexaprenyltranstransferase activit. . .	2	3
GO:0080030	methyl indole-3-acetate esterase activit. . .	2	4
GO:0003958	NADPH-hemoprotein reductase activity	2	5
GO:0003988	acetyl-CoA C-acyltransferase activity	2	6
GO:0005549	odorant binding	2	7

**Fig 6:**
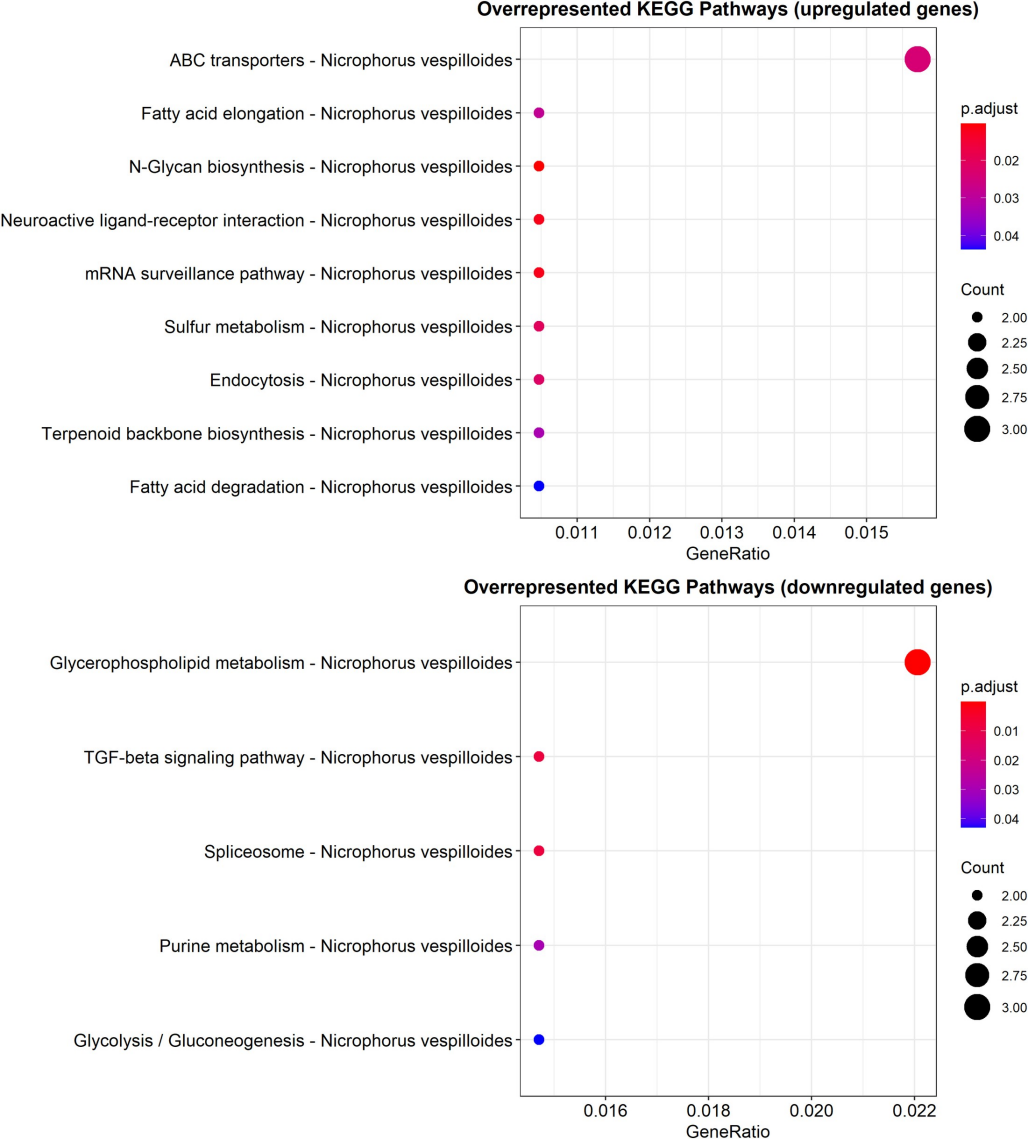
Enriched KEGG Pathways in the DEGs.

Identification of KEGG pathways activated or deactivated in fed insects compared to starved beetles was done using clusterProfiler [48]. The KEGG Orthology (KO) terms associated to the DEGs were compared to the KO terms assigned to *N*. *vespilloides*. The GSEA did not produce any enriched pathway. Instead, a term enrichment analysis was done using the same software package (Fig 6).

## Discussion

*Nicrophorus pustulatus* is considered unique among other burying beetles [15]. This species provides its brood with the same biparental care as other species of burying beetles and aggressively protects its offspring [16, 19]. However, it appears to have undergone a host shift, using snake eggs rather than vertebrate carcasses as a resource for its breeding [15–17]. Nonetheless, it still retains the ability to use vertebrate carcasses for reproduction if needed [15].

During the annotation of the RNA-seq derived transcriptome of *N*. *pustulatus* against the RefSeq database, most of the BLAST hits (>75%) were against reference sequences from *N*. *vespilloides*, which is presently the only *Nicrophorus* beetle with a genome assembly [50]. Additional BLAST hits identified transcripts more similar to other Coleoptera species such as the red flour beetle, *Tribolium castaneum*, and the dung beetle, *Onthophagus taurus*. These transcripts represent genes not present in *Nicrophorus vespilloides* RefSeq assembly and may either be incomplete or misassembled in the *N*. *vespilloides* RefSeq assembly or species-specific *N*. *pustulatus* genes.

Some assembled transcripts produced high scoring BLAST hits aligning to mite sequences, suggesting the potential presence of phoretic mites in *N*. *pustulatus* [51, 52]. Mites are highly prevalent in burying beetles populations and may be present in as many as 95% of individuals in a given population. Different species of phoretic mites, such as those from the genus *Uroobovell* and *Poecilochirus* have been shown to associate with *Nicrophorus* beetles [53, 54]. In addition, roughly 8% of the BLAST hits were against sequences from different species of bacteria (e.g. *Paenibacillus* sp., *Bacillus thuringiensis*, *Pseudomonas aeruginosa*, *Acinetobacter baumannii*, *Klebsiella pneumoniae*, and *Escherichia coli*) which contributes information about the microbiome of *N*. *pustulatus* and its environment. Core microbiota are transmitted vertically in burying beetles via the secretions that coat the carcass and inoculate feeding larvae [55], thus explaining the presence of bacterial-derived transcripts in the salivary glands of *N*. *pustulatus*. The presence of these transcripts in the current *N*. *pustulatus* transcriptome is similar to contaminant sequences found in the transcriptome assemblies of other Coleoptera species annotated using BLAST and similar databases. Roughly 10% of the hits obtained during transcriptome annotation of the seed beetle, *Callosobruchus maculatus*, belonged to viruses, bacteria or fungi (Sayadi, et al., 2016). The annotation of the Colorado Potato Beetle, *Leptinotarsa decemlineata*, produced 16% of BLAST hits outside of the Arthropoda [56, 57]. Dissections and RNA extractions were performed with the utmost care to prevent external contamination and mites were not found within the salivary gland tissue at the time of dissection. We do not have any evidence that these transcripts represent non-biological contamination. As such, these transcripts were not removed from the data used for downstream analysis and likely represent an accurate snapshot of the beetle’s gene expression.

Analysis of gene expression in *N*. *pustulatus* identified a total of 651 differentially expressed genes in fed beetles. Of them, 261 genes were downregulated and 390 genes were upregulated in fed beetles compared to the starved ones. While, perhaps unsurprising, both male and female beetles had a similar response indicating that the sex of the beetle did not alter the feeding related transcriptional alterations we investigated.

Several of the transcripts upregulated in fed insects compared to starved, were determined to encode hexamerin-like and arylphorin proteins (Fig 4). These two proteins belong to the same protein superfamily and are present in almost all insect species [58]. Hexamerin and arylphorin proteins are involved with the storage of energy and amino acids and also have a role in the insect immune response [59]. Similar to our findings, transcripts encoding hexamerin and arylphorin proteins were also downregulated in starved *Galleria mellonela* [60] and *Tribolium castaneum* [61].

Other genes involved in the insect immune response found to be upregulated in fed beetles during the quantitative analysis of the beetle transcriptome included defensin and serine proteases, including the circulative protease persephone. These genes are associated with the Toll and Imd signaling KEGG Pathway. Defensins are small, disulfide-rich peptides shown to exhibit a broad spectrum of antimicrobial activity against different parasites such as fungi and bacteria [62, 63]. These peptides have been previously found to be present in the oral and anal secretions of *N*. *vespilloides* [12] and transcripts encoding these peptides were upregulated in *N*. *vespilloides* challenged with heat inactivated bacteria [64], adult *N*. *orbicollis* [21], and the ticks *Haemaphysalis flava* [65] and *Amblyoma sculptum* [66] during blood feeding.

Serine proteases hydrolyze peptide bonds, making them a critical component of a number of biological processes, including digestion and immune response. Protein catabolism is integral to the digestion and usage of carrion, an incredibly protein-rich resource. In addition, serine proteases are a component of one of the most important processes in the innate immune responses of insects, the melanization process [67]. Serine protease upregulation has been reported in *N*. *vespilloides* that are breeding [20] and actively providing parental care [19]. A number of serine proteases specifically tied to immune response were identified in *N*. *vespilloides* challenged with heat inactivated bacteria [64]. These immunity-linked serine proteases included coagulation factor proteases, prophenoloxidase activating enzymes, and hemolymph proteases [62]. The circulative protease persephone has been found activate the Toll signaling pathway in *Drosophila* [68], and it is likely this protein has a similar role in *N*. *pustulatus*. The ability of persephone to sense a broad range of microbe virulence factors may enable *N*. *pustulatus* to launch immune responses against the diverse community of microbes present in soil and carcass microbiomes [63].

Other transcripts upregulated in the fed beetles were identified as Cytochrome P450 (CYP450). Insect CYP450s have many metabolic roles including the detoxification of several chemicals considered harmful for insects such as plant secondary metabolites and insecticides [69]. Transcripts encoding CYP450 were upregulated in *N*. *vespilloides* actively providing parental care [19], induced in *T*. *castaneum* following immunization [70], and induced in *Protaetia brevitarsis sulensis* during bacterial infection [71]. It has been hypothesized that the ability of CYP450 to produce reactive oxygen species leads to the initiation or amplification of the immune response [72].

A previous study showed that oral secretions in *N*. *pustulatus* lack antimicrobial activity [5], and in this study only one transcript encoding an antimicrobial peptide (AMP), defensin, was found to be upregulated in fed beetles. Our results suggest that *N*. *pustulatus* does not seem to upregulate genes in the salivary glands that encode AMPs following feeding. However other genes associated with the insect immune response as well as detoxification processes did increase their expression, maybe with the aim of lowering the detrimental effects that the presence of exogenous microbes have to their reproductive success and larval growth [6]. Opening snake eggs to allow feeding by larvae or burying a carcass to prepare a brood ball exposes feeding adults and larvae to soil and carrion microbes which may trigger immune response [52].

This work provides a snapshot of the transcriptional alterations that occur in the salivary glands of *N*. *pustulatus* following feeding and also provides a well described transcriptome for the further analysis of this unique burying beetle. The diet presented to beetles during this study was significantly different than food resources available for beetle in their native environment. As such, additional research simulating field-like conditions is warranted to fully understand the changes in gene expression of *N*. *pustulatus* during its usual reproduction cycle, which will show for sure a different expression profile than the one reported by this work. Nonetheless, additional studies are underway to compare and contrast the alterations in the salivary glands of *N*. *pustulatus* following feeding with other burying beetle species.
